# Convergence research and training in computational bioengineering: a case study on AI/ML driven biofilm-material interaction discovery

**DOI:** 10.21203/rs.3.rs-3318640/v1

**Published:** 2023-09-08

**Authors:** Jessica LS Zylla, Etienne Gnimpieba Z., Alain B Bomgni, Rajesh K Sani, Mahadevan Subramaniam, Carol Lushbough, Robb Winter, Venkataramana R Gadhamshetty, Parvathi Chundi

**Keywords:** Convergence research and training, Biofilm engineering, Material science, Data science, Artificial intelligence, Machine learning

## Abstract

Initially, research disciplines operated independently, but the emergence of trans-disciplinary sciences led to convergence research, impacting graduate programs and research laboratories, especially in bioengineering and material engineering as presented here. Current graduate curriculum fails to efficiently prepare students for multidisciplinary and convergence research, thus creating a gap between the students and research laboratory expectations. We present a convergence training framework for graduate students, incorporating problem-based learning under the guidance of senior scientists and collaboration with postdoctoral researchers. This case study serves as a template for transdisciplinary convergent training projects - bridging the expertise gap and fostering successful convergence learning experiences in computational biointerface (material-biology interface). The 18-month Advanced Data Science Workshop, initiated in 2019, involves project-based learning, online training modules, and data collection. A pilot solution utilized Jupyter notebook on Google collaborator and culminated in a face-to-face workshop where project presentations and finalization occurred. The program started with 9 experts in the four diverse fields creating 14 curated projects in data science (Artificial Intelligence/Machine Learning), material science, biofilm engineering, and biointerface. These were integrated into convergence research through webinars by the experts. The experts chose 8 of the 14 projects to be part of an all-day in-person workshop, where over 20 learners formed eight teams that tackled complex problems at the interface of digital image processing, gene expression analysis, and material prediction. Each team was comprised of students and postdoctoral researchers or research scientists from diverse domains including computer science, materials science, and biofilm research. Some projects were selected for presentation at the international IEEE Bioinformatics conference in 2022, with three resulting Machine Learning (ML) models submitted as a journal paper. Students engaged in problem discussions, collaborated with experts from different disciplines, and received guidance in decomposing learning objectives. Based on learner feedback, this successful experience allows for consolidation and integration of convergence research via problem-based learning into the curriculum. Three bioengineering participants, who received training in data science and engineering, have received bioinformatics jobs in biotechnology industries.

## Challenge Statement

We posited the question of whether bioengineering students, particularly graduate students, were prepared for laboratory work in convergence science? The convergence science approach combines multiple fields to solve a problem where teammates may come from very distinct fields (e.g., computer science, biological sciences, engineering) [1]. The rise of transdisciplinary sciences has exposed skill gaps among graduate students. The students are entering the workforce from laboratories that likely did not participate in convergent research and lack the skills to communicate across diverse research disciplines. This gap in their education prompted us to adopt a dual strategy: utilizing a convergence science focus with project-based learning.

**Figure 1:**
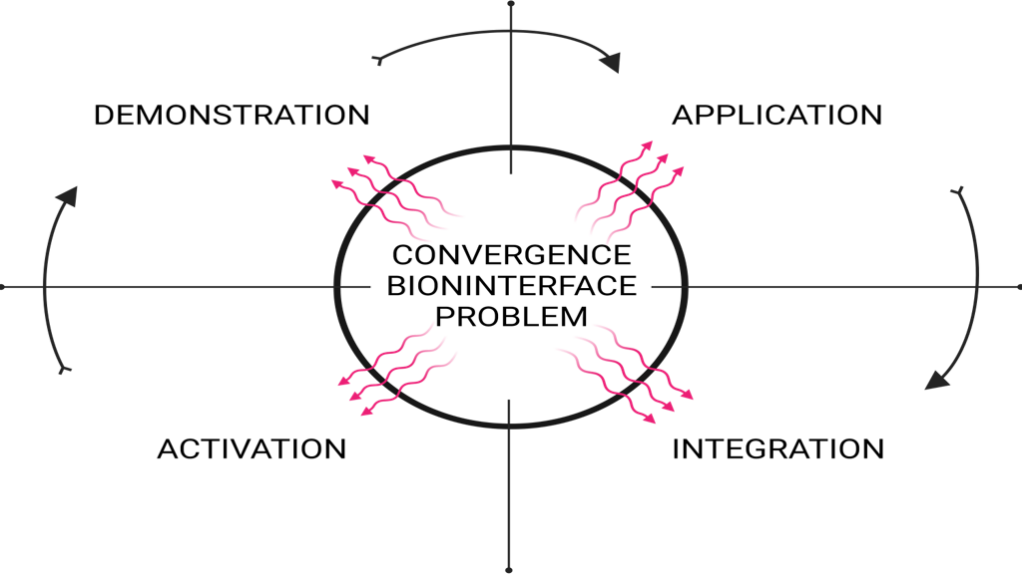
Merrill’s first five principles of instruction. Adapted from Merrill 2002, Created with BioRender.com

We present a specialized convergence framework designed for graduate students to bridge this gap. This project-based learning model uses the five first principles from Merrill 2002 [2] Fig. 1. This innovative approach intertwines problem-based learning with seasoned scientists’ mentorship and postdoctoral researchers’ collaboration utilizing the convergence research approach in each of the five areas (Fig. 1). By following this model, we provide a blueprint for other transdisciplinary projects. This initiative successfully bridges the expertise gap and nurtures effective convergence learning experiences within the realm of computational biointerface (material-biology interface). This guidance by experts in the field allows students’ previous experience to guide their input as they develop transdisciplinary skills.

## Novel Initiative

Our case study in this manuscript addresses the unpreparedness for multidisciplinary and convergent research of bioengineering graduate students that results in a gap between their skills and research laboratory expectations. The aim is to establish a comprehensive synthetic framework capable of addressing intricate scientific and societal challenges at the crossroads of diverse domains.

**Figure 2:**
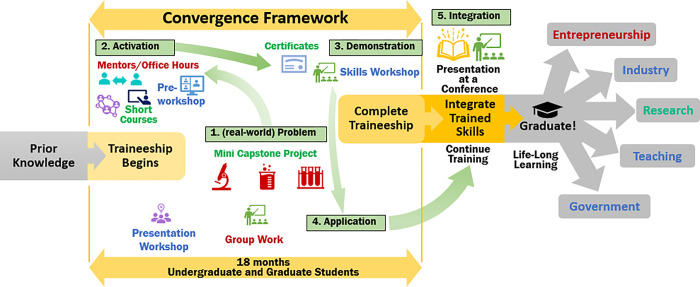
DDMD Advanced Data Science Workshop Framework. This figure explains how the many facets of the DDMD workshop lean into Merrill’s first five principles and lead graduates to perform convergent research and graduate the workshop and/or their program and go into any career type they choose. This framework leads to convergent research artifacts that prepare students for continued real-world applications.

Our framework (Fig. 2) involved assembling experts across three disciplines – microbiology, material sciences with a focus on 2D materials, and big data – to engage in interdisciplinary ventures (Fig. 1). This collaboration facilitated productive dialogues between computer science and biology students, enhancing mutual comprehension and enriching their understanding of each other’s fields. To bolster competencies, we embraced portions of the Pedagogy-Andragogy-Heutagogy (PAH) continuum [3] – a combination methodology empowering learners to use firsthand experiences for deeper understanding while having a locus of control that is still teacher -learner with the expert guiding the team, —focusing on problem-solving into a graduate education convergence framework. Culminating in the students’ participation in transdisciplinary projects.

This solution centers on enhancing students’ aptitude for interdisciplinary teamwork and aligning with research laboratory standards. By integrating 2D material science with computational biointerface, we introduced a convergence framework for graduate students. This framework, guided by senior scientists and postdoctoral researchers, embraces problem-based learning and the first principles of instruction by Merrill (2002).

## Development and Implementation

### Convergence Problem and Team Science

The framework, which we referred to as the DDMD (Data Driven Material Discovery) Advanced Data Science Workshop, incorporates Merrill’s first five principles [2]. The complete program spanned 18 months (about 1 and a half years) and included numerous meetings, workshops, and dedicated office hours (Fig. 2). The framework combined team projects, project-based learning, online training modules, and data collection for workshop improvement, utilizing surveys during each step. Through this platform, we engaged their existing knowledge with short courses, certificates, and mentorship, which cultivated collaboration, to enhance the second principle. We took students from their previous knowledge and expanded their knowledge to improve their interdisciplinary skillset. We promoted learning by enabling participants to demonstrate, apply, and integrate their new knowledge [2]. This was done by assuring that objectives were aligned across the discipline skills learned [4].

### Mini-Capstone Projects and Expert selection and Group Selection

Merrill’s first of the first five principles (2002) asserts that problems pertinent to the real-world are most impactful to effective learning. We used this to our advantage when developing the mini-capstones for this framework. The program started with 9 experts in four fields creating 14 curated projects in data science (Artificial Intelligence/Machine Learning), material science, biofilm engineering, and biointerface (Table 1). These were integrated into convergence research through webinars by the experts. Participants, including experts from diverse fields, formed teams and tackled subjects such as digital image processing, gene expression analysis, and material prediction.

Out of the 14 projects (Table 1), the experts, working with the participants, selected eight to be included in the future all-day in-person workshop, based on their interest. During this event, more than 20 learners formed eight teams to tackle topics such as digital image processing, gene expression analysis, and material prediction. Each team was composed of students and a postdoctoral researcher or scientist representing each related scientific field (computer, material, biofilm). Organizing the groups this way facilitated a transition from initial skillsets to a convergence skillset.

### Online Training with Short Courses, Office Hours, and Pre-Workshop Meeting

From the selected eight projects, the field experts determined the base knowledge required by the participants and curated short courses accordingly. Each short course included topic specific learning objectives. This allowed us to activate participants’ prior knowledge as a foundation for new knowledge (Merrill’s second principle, Merrill 2002). The experts used Jupyter Notebook and Google Colab to reduce barriers of access for the participants. The short courses were housed in a Google Classroom for the graduate and undergraduate students to go through at their own pace. Each mini-capstone project had a project outline document describing the science problem followed by computational tasks to address them. The participant also had access to a template notebook as project programming playground on toy dataset (S1.zip). An orientation workshop to these modules allowed the students to choose theprojects to join for the remaining workshops and office hours.

Our short courses consisted of nine topics, and we kept the courses available to the participants for 6 months after the workshop conclusion. These courses gave the participants background knowledge of the topics within all the projects. Given the convergent nature of these projects, it was essential for the groups to meet and collaboratively address questions. Therefore, the small groups met twice during the interim times between workshops. Each project was required to have 3 office hours led by the project expert. A 2-hour office hour introduction to the project and a subsequent 1-hour work time and question/answer session office hour were held.

### Certificates and Short Course Workshop

Merrill’s third principle is that to promote learning new knowledge must be demonstrated by the learners. Our experts implemented certificates for participants that finished their courses. The certificates obtained from the short courses demonstrated the expansion of the student’s existing knowledge, allowing students to improve their skillset through the acquisition of new interdisciplinary subjects.

### In-Person Workshop, Group Work, and Presentations

The comprehensive process of project creation, team formation, equipping participants with skillsets for project engagement, culminated in an in-person workshop where project presentations and finalization occurred. This approach facilitated ongoing leaning for participants, as promoted through application of new knowledge, Merrill’s fourth principle [2].

For our final meeting we had an in-person workshop, with attendees able to join online viz Zoom. First half of the day one discipline type presented their projects to the whole workshop. Subsequently, we allocated time for collaborative group work, during which time students presented their projects’ status. Upon completion of this workshop, students had opportunities to ask questions and continue working on their projects with their teams.

### Artifacts and Conference(s)

This framework produced artifacts from each mini-capstone project. The various projects had artifacts produced during the 18 months, with IEEE presentations in both 2021 and 2022 conference proceedings [5–13]. Performance in the production of these artifacts (presentations, machine learning models) allowed the participants to integrate their new knowledge (Merrill’s 5th principle) [2] into a presentation at the conference. Three machine learning models were created.

We distributed via email pre and post workshop forms, as well as a small group form meant for participants of the office hours. We asked questions pertaining to relevant convergence team building data (e.g. their DDMD role (academic position), previous experience with various skills, goals) and we asked for feedback on what participants thought what went well and desired improvements. Out of our 22 participants, we had various amounts of responses to the surveys given, as detailed in the reflection section.

## Reflection

### Short Course Workshop

Following the short course workshop participants rated (1 to 4, 1 low and 4 high) the quality of six workshop facets via survey. The following table displays frequency distributions of recorded ratings and mean ratings (Table 2).

Participants appreciated the short course workshop for its comprehensive information and organized approach. The overviews of various tasks proved invaluable for navigating the upcoming month, and the structured presentation of separate projects was particularly enlightening. Learning about module expectations and the mini-capstone project’s scope eased concerns and heightened enthusiasm for the course. The professors’ consistent reassurance, encouragement of questions, and ongoing support were highly valued, as was the chance to delve into machine learning content and work on a capstone project. The workshop’s well-structured divisions and implementation sparked excitement for upcoming mini-capstone projects and advanced data science exploration. The Python and machine learning modules were singled out as exceptional resources, especially appreciated by those seeking practical skills. The workshop was well received but we did find comments that the activation activities (Fig. 1) could have been shorter.

### Small Groups and Office Hours

To gauge the effectiveness of our small groups’ work we surveyed the participants. The data shows good outcomes, but due to the small size of the group, the results do not allow robust interpretation (n = 4). We will explore this dimension in our future cohorts.

### In-person Workshop in Montana

**Figure 3:**
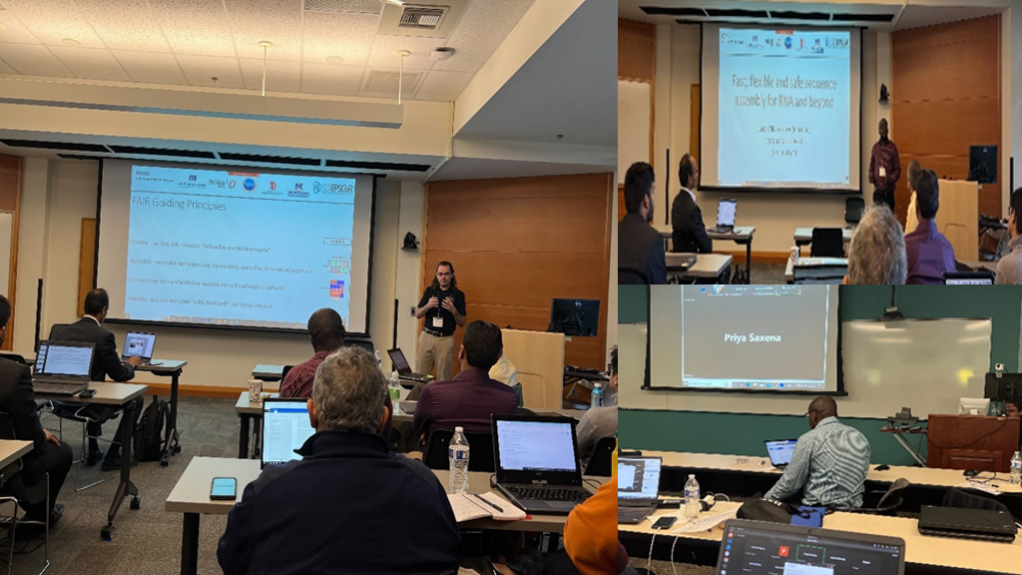
Pictures of the in-person Workshop in Montana.

This experience culminated in an in-person workshop in Montana where we started with capstone presentations and then gave the student participants the opportunity to present the project’s computer science background. We had participants in person and online via Zoom (Fig. 3). We found it was easy to portray the skill information via Zoom since most of the participation was through the short courses.

There were a total of 22 participants in the mini-capstones that responded to a survey. The overall quality ratings are displayed below disaggregated by the five DDMD (Data Driven Material Discovery) participation roles (Table 4). Recorded ratings appeared to be independent of DDMD role. The mean quality rating was 9.26. There were no responses lower than 7; therefore, we omitted them in Table 4.

The October 19th workshop had five learning objectives as seen in Table 5. Participants rated the extent (1 to 5, 1 = not at all, 2 = limited extent, 3 = moderate extent, 4 = substantial extent and 5 = exceptional extent) to which they felt each of the five objectives were attained. Mean ratings and frequency distributions of recorded ratings are displayed in the following table for each of the five objectives.

Respondents rated the quality (1 to 4, 1 = low quality and 4 = high quality) of four workshop facets. “I didn’t do this” was an available response which two respondents selected for “consultations with experts.” Mean ratings and numbers of recorded ratings are displayed in the following table (Table 6).

Participants highlighted several workshop strengths, including bridging the gap between biology and computation, promoting collaboration across disciplines, fostering communication and involvement, facilitating interaction between subject matter experts and data scientists, emphasizing the multidisciplinary nature of the workshop, providing high-quality instructions with active participant engagement, offering insights into various capstone projects, showcasing state-of-the-art technologies relevant to participants’ future professions, and creating a comfortable and collaborative environment conducive to learning and questioning. The workshop’s value was also recognized in terms of understanding the role of machine learning in addressing diverse challenges and the opportunity for one-on-one and group discussions. Based on participants’ views on the strength of this workshop we accomplished our goal of promoting collaboration and the students’ skillset in interdisciplinary convergence science.

### Feedback on Workshop Improvement

Participants provided insightful suggestions for improving the workshop experience. Two respondents advocated for in-person attendance to address time management issues linked to virtual participation (proposed image montana Fig. 3). Another recommended increased engagement for online participants and improved audio/visual components to enhance both in-person and virtual aspects. Four participants sought more time for team collaboration, discussions, and programming tasks. Other suggestions included adhering more closely to the agenda, offering greater background on projects and coding possibilities, improving presentation organization, and providing more meaningful data for machine learning training. Reducing the number of projects was proposed to enable deeper exploration, using more robust datasets.

For summative evaluation purposes, 21 respondents retrospectively recorded pre- and post-workshop participation ratings from 1 to 4 (1 = low and 4 = high) of their understanding of what it takes (workflow) to harvest data from a variety of sources to address a specific question.

The paired dependent-t test was used to test for pre/post mean differences at the 0.05-level of significance with null hypothesis: difference = 0 and alternative hypothesis: difference < > 0. Mean rating ( ), standard deviation (SD), matched pair dependent t-statistic (t), p-value (p), the correlation between the matched pair ratings, and effect size^1^ are displayed in the following table. The increase in mean ratings of understanding from pre- to post-was statistically significant (t = 3.35, p < 0.0032) and the effect size was medium (0.72).

**Figure 4:**
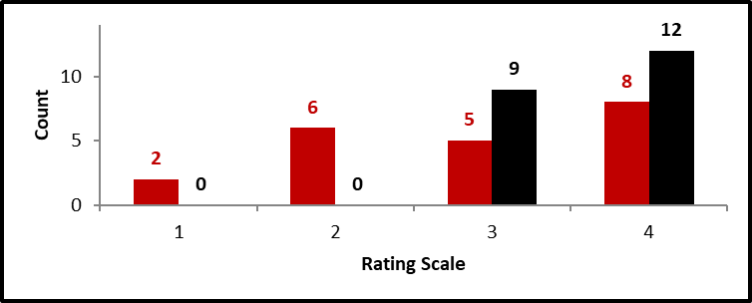
Participants’ view of their understanding of what it takes to harvest data from a variety of sources to address a specific question. pre- and post-workshop.

We performed analysis to examine the participants’ view of their understanding of what it takes to harvest data from a variety of sources to address a specific question. pre- and post-workshop (Fig. 4). The **deep red bars** represent pre-workshop ratings and **black bars** represent post-workshop ratings.

### Pre-Post Ratings of Workflow Understanding

The outcome exceeded expectations: some projects earned slots for presentation at the international IEEE Bioinformatics conference in 2022 [5–8], yielding three Machine Learning models published in a journal [9–18]. Feedback from participants affirmed the success of this experience, underlining the potential for integrating convergence research into the curriculum through problem-based learning. Moreover, the initiative yielded tangible results, with bioengineering graduate students, trained in data science and engineering, securing positions in the biotechnology sector.

The success of our framework in fostering convergence science teams to address transdisciplinary challenges holds promise. Implementing this model in both undergraduate and graduate studies could shape the next generation of researchers. By championing collaboration, problem-solving, and integration of convergent research methodologies, we pave the way to bridge knowledge gaps, prepare our students for convergence science and propel scientific innovation.

## Figures and Tables

**Figure 5:**
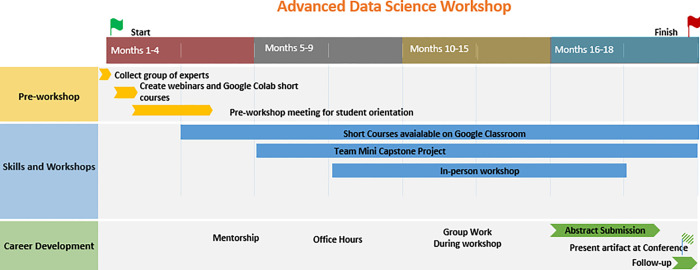
Gantt View of the Advanced Data Science Workshop Framework

**Table 1:** Mini-Capstone Projects

Pilot Projects:	Projects under development:
P001 Essential gene prediction	P008 Biocorrosion Prediction
P002 Gene Name Entity Recognition	P009 Complex image analysis in Bio-Interface
P003 Bacteria Cell Segmentation	P010 Multi-OMICS driven Biomarker discovery in Biointerface
P004 Image Classification using Deep learning	P011 Material Dimension prediction
P005 Data visualization	P012 Genomic data decomposition (ICA, GRN inference)
P006 Antimicrobial Peptide Prediction	P013 Composition based Material property prediction
P007 Nanowire Protein Prediction	P014 Mutant Prediction/classification

**Note**: P001-P008 denote the 8 selected projects for an in-person workshop because chosen by participants

**Table 2:** Quality of Six Workshop Facets

	Mean Rating	Number of Recorded Ratings			
		1 = low	2	3	4 = high
The helpfulness of the information about the workshop that you received prior to today.	3.31		3	3	7
The overall organization of today’s event.	3.61		1	3	9
The use of your time today.	3.61			5	8
The clarity of expectations of workshop participants.	3.54		2	2	9
The opportunities today that were provided for participants to ask questions.	3.92			1	12
The process for assigning the teams for the capstone projects.	3.67		1	2	9

**Table 4:** 

DDMD Role	Table 4: Ratings of the Quality of the Capstone Project Experience				
	7	8	9	10	
Early-career researcher			1	1	2
Postdoc or Research Assistant	1			4	5
Master’s Graduate Student		1	2	1	4
PhD Graduate Student		1	2	3	6
Undergraduate		1		1	2
	1	3	6	10	20

**Table 5:** Extent of the Attainment of Workshop Learning Objectives

Objective	Mean	Number of Recorded Ratings				
		1	2	3	4	5
Explore the cutting-edge of modern biology and material informatics tools, including machine learning, data analysis and visualization, and molecular/multiscale modeling.	4.29			4	7	10
Learn how to fine-tune general-purpose models for bioscience and material applications.	4.18		1	4	7	10
Learn how to work with small, sparse, or low-quality datasets and build predictive models.	4.36			4	6	12
Deepen your knowledge of the frontiers of data-driven MOICs and material analysis and ready-to-deploy code solutions.	4.05		1	6	6	9
Learn computational methods and codes for building better materials, such as language models, protein models and graph neural networks, and how to build and use your own custom datasets.	4.38		1	3	4	13

**Table 6:** Workshop Quality

Description	Mean	Number of Recorded Ratings			
		1 = low quality	2	3	4 = high quality
The mini capstone project presentations	3.67			7	14
The programming material presentation	3.67			7	14
The group project	3.71			6	15
Consultations with experts	3.74		1	3	15

**Table 7:** Dependent t-test Statistics (N = 21)

Statement	Pre		Post		r	t	p	Effect size
	□	SD	□	SD				
Your understanding of what it takes (workflow) to harvest data from a variety of sources to address a specific question.	2.90	1.04	3.57	0.51	0.49	3.35	0.0032	0.72

**Table 8:** **Summary process** Below are the steps to implement in Undergraduate and Graduate Bioengineering studies, summer project planning and completion taking about 18 months (Fig. 5).

**Step 1.**	**Choose Faculty and Postdoctoral Researchers (the experts) to lead projects**
Step 2.	Initiate a brainstorming session and allow the experts choose their projects
Step 3.	Each expert creates baseline material and puts in a curated Google Classroom
Step 4.	Have a conference call to orient students in the Google Classroom
Step 5.	Give the students time to participate on the Google Classroom
Step 6.	Combine students in their chosen projects via a skills workshop
Step 7.	Hold office hours between workshops
Step 8.	Have conference where students present as the ‘other’ discipline
Step 9.	Students complete their mini capstones, creating an artifact that they can present at a conference

## References

[1] Committee on Key Challenge Areas for Convergence and Health, Convergence: Facilitating Transdisciplinary Integration of Life Sciences, Physical Sciences, Engineering, and Beyond. National Academies Press (US), 16 June 2014. doi:10.17226/18722 24830066

[2] Merrill M. David. “First Principles of Instruction.” Educational Technology Research and Development, vol. 50, no. 3, 2002, pp. 43–59. JSTOR, http://www.jstor.org/stable/30220335. Accessed 10August 2023.

[3] Ekoto Christian Eugene, Gaikwad Prema. The Impact of Andragogy on Learning Satisfaction of Graduate Students. American Journal of Educational Research. Vol. 3, No. 11, 2015, pp 1378–1386. http://pubs.sciepub.com/education/3/11/6

[4] Sharp Phillip, “Capitalizing on Convergence for Health Care.” Science, vol. 352, no. 6293, 2016, pp. 1522–23. JSTOR, http://www.jstor.org/stable/24747486. Accessed 1 Sept. 2023. 27339972 10.1126/science.aag2350

[5] Bomgni A.B., Fotseu E.B.F., Wambo D.R.K., Sani R.K., Lushbough C. and Zohim E.G., 2022, December. Attention model-based and multi-organism driven gene recognition from text: application to a microbial biofilm organism set. In 2022 IEEE International Conference on Bioinformatics and Biomedicine (BIBM) (pp. 3596–3598). IEEE. doi: 10.1109/BIBM55620.2022.9995269.

[6] Haas S., Hartman T.W., Gurung B.D.S., Do T., Sani R., Gadhamshetty V. and Gnimpieba E., 2022, December. Using BASIN-ML for Machine Learning-Based Statistical Analysis and Reporting for Biofilm Datasets. In 2022 IEEE International Conference on Bioinformatics and Biomedicine (BIBM) (pp. 3605–3607). IEEE. doi: 10.1109/BIBM55620.2022.9995185.

[7] Gurung B. D. S. Devadig R. Do T. Gadhamshetty V.and Gnimpieba E. Z. “U-Net Based Image Segmentation Techniques for Development of Non-Biocidal Fouling-Resistant Ultra-Thin Two-dimensional (2D) Coatings,” 2022 IEEE International Conference on Bioinformatics and Biomedicine (BIBM), Las Vegas, NV, USA, 2022, pp. 3602–3604, doi: 10.1109/BIBM55620.2022.9995609.

[8] Singh R. N. Gnimpieba E. Z. and Sani R. K. “Title: Challenges in single cells sequencing Microbial community and biofilm: A case of Oleidesulfovibrio alaskensis G20 NGS protocol,” 2022 IEEE International Conference on Bioinformatics and Biomedicine (BIBM), Las Vegas, NV, USA, 2022, pp. 3613–3615, doi: 10.1109/BIBM55620.2022.9995610.

[9] Thakur P. , “Identifying genes involved in biocorrosion from the literature using text-mining,” 2021 IEEE International Conference on Bioinformatics and Biomedicine (BIBM), Houston, TX, USA, 2021, pp. 3586–3588, doi: 10.1109/BIBM52615.2021.9669354.

[10] Tripathi A. K. , “Discovery of genes associated with sulfate-reducing bacteria biofilm using text mining and biological network analysis,” 2021 IEEE International Conference on Bioinformatics and Biomedicine (BIBM), Houston, TX, USA, 2021, pp. 3589–3591, doi: 10.1109/BIBM52615.2021.9669374.

[11] Fotseu E. B. F. Nembot T. K. Sani R. K. Gadhamshetty V. Gnimpieba Z. Etienne and Bomgni, A. B. “GenNER - A highly scalable and optimal NER method for text-based gene and protein recognition,” 2021 IEEE International Conference on Bioinformatics and Biomedicine (BIBM), Houston, TX, USA, 2021, pp. 3562–3569, doi: 10.1109/BIBM52615.2021.9669827.

[12] Nembot T. K., Fotseu E. B. F. Sani R. K., Gnimpieba Z. Etienne Lushbough C. and Bomgni A. B. “Prediction of essential genes in G20 using machine learning model,” 2021 IEEE International Conference on Bioinformatics and Biomedicine (BIBM), Houston, TX, USA, 2021, pp. 3578–3582, doi: 10.1109/BIBM52615.2021.9669756.

[13] Gasper W. Ma J. Ghersi D. Gnimpieba E. Z. Gadhamshetty V. and Chundi P. “Automatic Extension of Medical Subject Headings (MeSH) Thesaurus to Emerging Research,” 2021 IEEE International Conference on Bioinformatics and Biomedicine (BIBM), Houston, TX, USA, 2021, pp. 3570–3577, doi: 10.1109/BIBM52615.2021.9669520.

[14] Thakur P, Alaba MO, Rauniyar S, Singh RN, Saxena P, Bomgni A, Gnimpieba EZ, Lushbough C, Goh KM, Sani RK. Text-Mining to Identify Gene Sets Involved in Biocorrosion by Sulfate-Reducing Bacteria: A Semi-Automated Workflow. Microorganisms. 2023;11(1):119. doi: 10.3390/microorganisms11010119. 36677411 PMC9867429

[15] Tripathi AK, Thakur P, Saxena P, Rauniyar S, Gopalakrishnan V, Singh RN, Gadhamshetty V, Gnimpieba EZ, Jasthi BK and Sani RK (2021) Gene Sets and Mechanisms of Sulfate-Reducing Bacteria Biofilm Formation and Quorum Sensing With Impact on Corrosion. Front. Microbiol. 12:754140. doi: 10.3389/fmicb.2021.754140 34777309 PMC8586430

[16] Raya D, Shreya A, Kumar A, Giri SK, Salem DR, Gnimpieba EZ, Gadhamshetty V and Dhiman SS (2022) Molecular regulation of conditioning film formation and quorum quenching in sulfate reducing bacteria. Front. Microbiol. 13:1008536. doi: 10.3389/fmicb.2022.1008536 36386676 PMC9659907

[17] Saxena P, Rauniyar S, Thakur P, Singh RN, Bomgni A, Alaba MO, Tripathi AK, Gnimpieba EZ, Lushbough C and Sani RK (2023) Integration of text mining and biological network analysis: Identification of essential genes in sulfate-reducing bacteria. Front. Microbiol. 14:1086021. doi: 10.3389/fmicb.2023.1086021 37125195 PMC10133479

[18] Allen C, Aryal S, Do T, Gautum R, Hasan MM, Jasthi BK, Gnimpieba E and Gadhamshetty V (2022) Deep learning strategies for addressing issues with small datasets in 2D materials research: Microbial Corrosion. Front. Microbiol. 13:1059123. doi: 10.3389/fmicb.2022.1059123 36620046 PMC9815019

